# Supplementary material for: EIF4A3-Mediated circ_0008126 Inhibits the Progression and Metastasis of Gastric Cancer by Modulating the APC/β-Catenin Pathway
Source: Cancers (Basel). 2025 Jan 14;17(2):253. doi: 10.3390/cancers17020253 (PMC11763408; doi:10.3390/cancers17020253)
Supplement: Supplementary file 1 [file cancers-17-00253-s001.zip › cancers-3302731-supplementary.pdf]

Supplementary Materials: EIF4A3-Mediated circ_0008126 Inhibits the Progression and Metastasis of Gastric Cancer by Modulating the APC/β-Catenin Pathway

Zeen Wang, Wenxing Chen, Ziwei Wang and Xinglong Dai

Table S1. Primers, Probes, and siRNA information.

Primers/Probes	Sequence
hsa_circ_0008126-F	5′-TCCACAAATCAGCTCGCCA-3′
hsa_circ_0008126-R	5′-CATCAAATTCTTTATCTGAGCAGC-3′
STX12-F	5′-AGAAAGAGAAACGGCAATTCGG-3′
STX12-R	5′-CCTGGTCATGGATCATCATGG-3′
GADPDH-F	5′-CTTTGGTATCGTGGAAGGACTC-3′
GADPDH-R	5′-GTAGAGGCAGGGATGATGTTCT-3′
APC-F	5′-AAAATGTCCCTCCGTTCTTATGG-3′
APC-R	5′-CTGAAGTTGAGCGTAATACCAGT-3′
EIF4A3-F	5′-GACTCTGAATCATGGCGACCA-3′
EIF4A3-R	5′-CAAAACCGTAAGCGTAGATGCC-3′
E-cadherin F	5′-AGTCACTGACACCAACGATAAT-3′
E-cadherin R	5′-ATCGTTGTTCACTGGATTTGTG-3′
Vimentin F	5′-GGACCAGCTAACCAACGACA-3′
Vimentin R	5′-AAGGTCAAGACGTGCCAGAG-3′
Ki-67 F	5′-CAGACATCAGGAGAGACTACAC-3′
Ki-67 R	5′-GTTAGACTTGCTGCTGAGTCTA-3′
PCNA F	5′-TAATTTCCTGTGCAAAAGACGG-3′
PCNA R	5′-AAGAAGTTCAGGTACCTCAGTG-3′
Si-circ_0008126#1	5′-CUACUUCAGAACAGCUGCUTT-3′
	5′-AGCAGCUGUUCUGAAGUAGTT-3′
Si-circ_0008126#2	5′-ACUUCAGAACAGCUGCUCATT-3′
	5′-UGAGCAGCUGUUCUGAAGUTT-3′
Si-circ_0008126#3	5′-ACAGCUGCUCAGAUAAAGATT-3′
	5′-UCUUUAUCUGAGCAGCUGUTT-3′
Si-EIF4A3#1	5′-UGAUCUGCUUGAUUGCUCGTT-3′
	5′-CGAGCAAUCAAGCAGAUCATT-3′
Si-EIF4A3#2	5′-UUCAUGUAGUCACCGAGAGTT-3′
	5′-CUCUCGGUGACUACAUGAATT-3′
Si-EIF4A3#3	5′-AUCUCAUCAAUCUGAGUGGTT-3′
	5′-CCACUCAGAUUGAUGAGAUTT-3′
FISH-circ_0008126 probe	5′-CTGAGCAGCTGTTCTGAAGTAGATA-3′
FISH-miR-502-5p probe	5′-TAGCACCCAGATAGCAAGGAT-3′
Pull down-circ_0008126 probe	5′-CTCCCACCTGAGCAGCTGTTCTGAAGTAGATACTT-3′

Table S2. The relationship between the expression of circ_0008126 and clinicopathological variables in 35 GC patients.

Patient Characteristics	Total	Circ_0008126 expression		P Value
		High	Low	
Age				0.686
≤65	22	12	10	
>65	13	8	5	
Gender				0.599
Male	24	13	11	
Female	11	7	4	
Tumour size(cm)				0.380
≤5	17	11	6	
>5	18	9	9	
TNM stage				0.031*
I/II	19	14	5	
III/IV	16	6	10	
Distant metastasis				0.167
No	28	19	12	
Yes	7	1	3	
Lymph node status				0.025*
Negative	17	13	4	
Positive	18	7	11	

The median circ_0008126 expression score (0.50) was defined as the cut-off value for dividing all GC patients into high- and low-expression groups. *$P<0.05$.

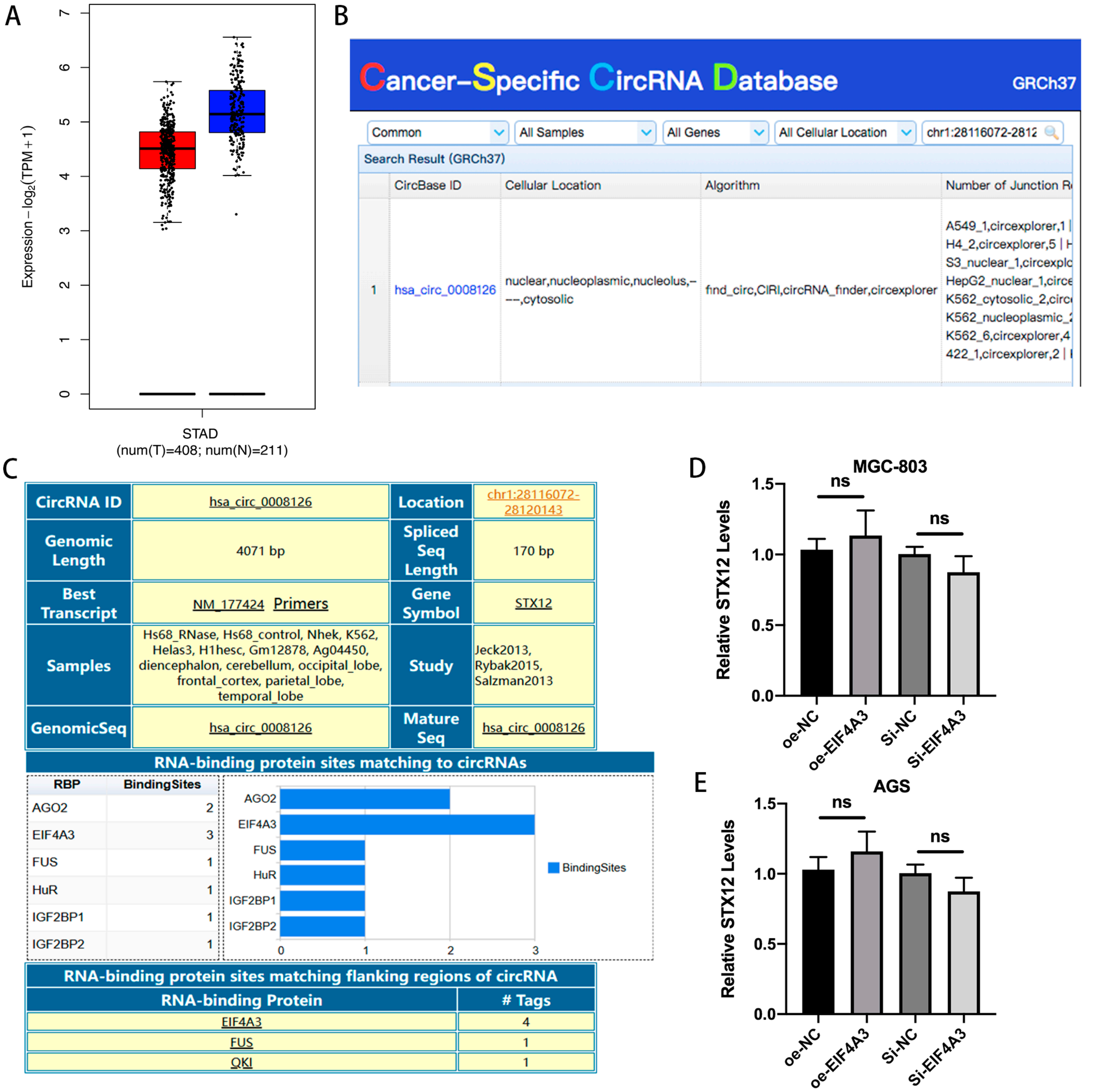

Figure S1. A. STX12 levels were detected in human GC normal tissues (n=211) and tumor tissues (n=408) by the TCGA database. B. The location and expression of circ_0008126 in the cytoplasm and nucleus are predicted by the CSCD database. C. The basic information and potential target molecules of circ_0008126 were predicted by the CircInteractome tool. D, E. The expression of STX12 in GC cells transfected with oe-NC or oe-EIF4A3 overexpression or Si-NC or Si-EIF4A3 was validated by qRT-PCR assays.

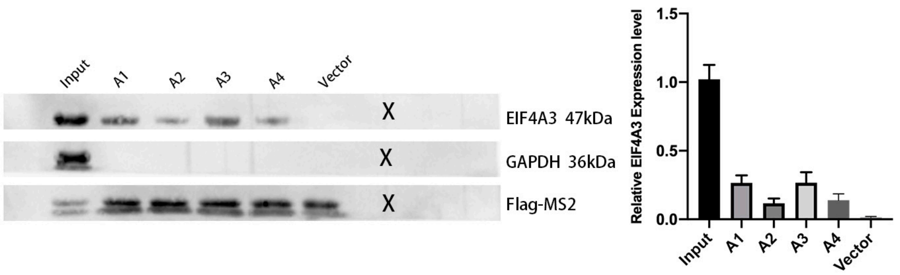

Figure S2. The protein levels were analyzed by the MS2 RIP assays.

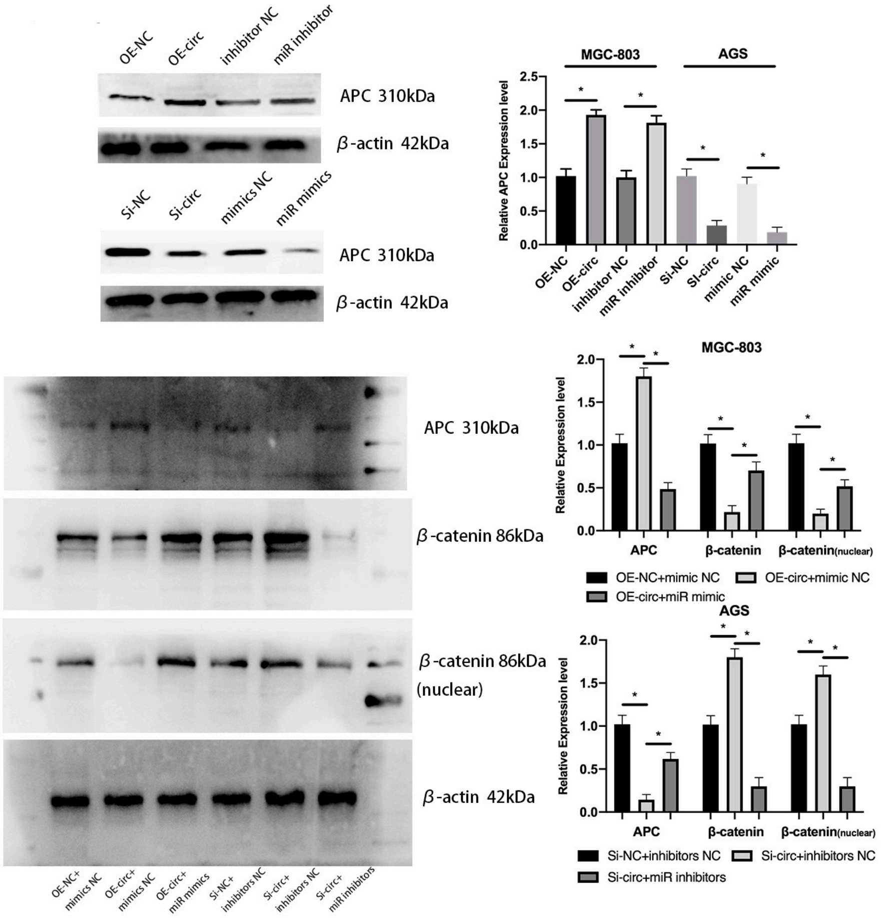

Figure S3. The original images and protein levels were measured by the western blots.

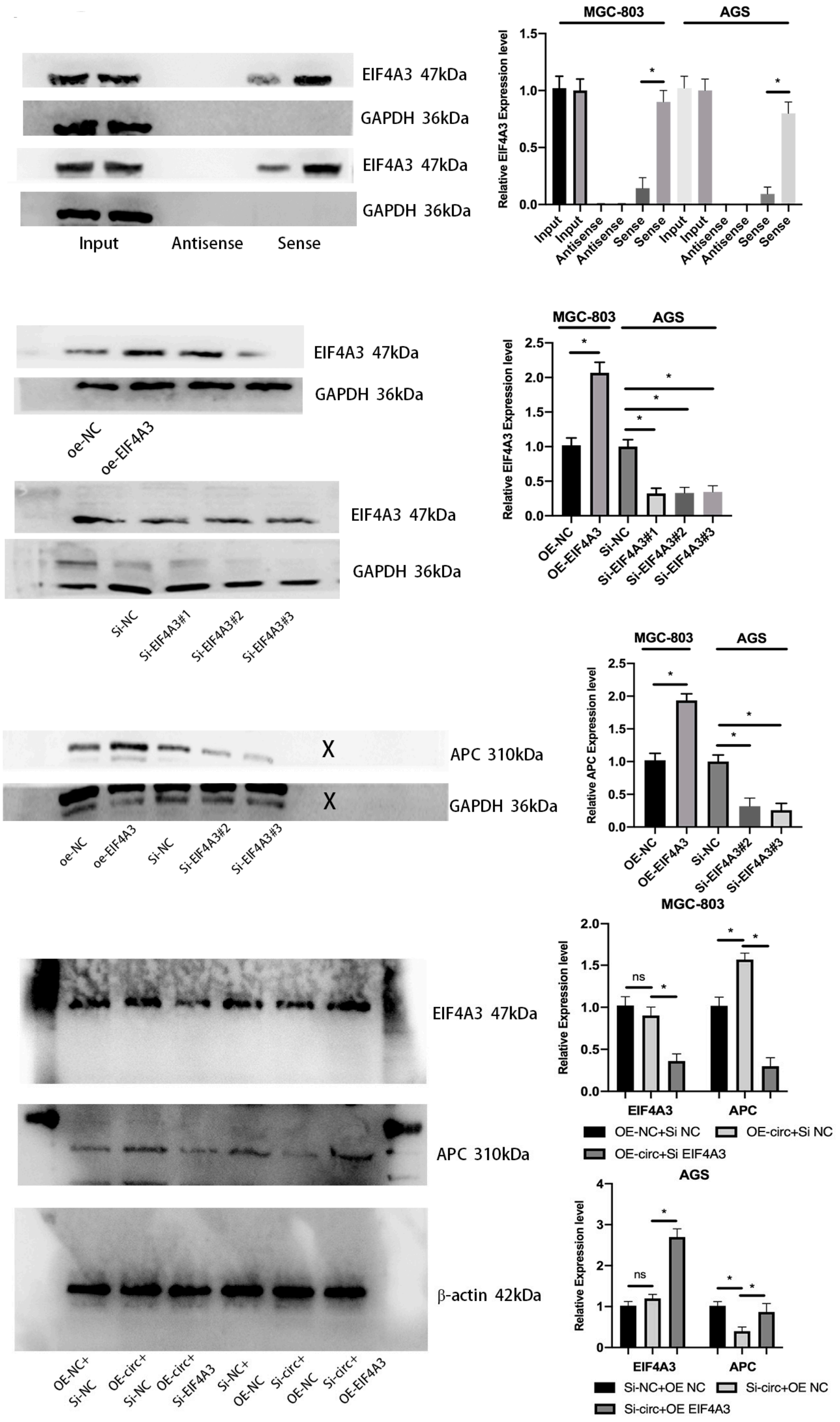

Figure S4. The original images and protein levels were measured by the western blots.
